# Survival with sildenafil and inhaled iloprost in a cohort with pulmonary hypertension: an observational study

**DOI:** 10.1186/s12890-015-0164-2

**Published:** 2016-01-12

**Authors:** Henning Gall, Natascha Sommer, Katrin Milger, Manuel J. Richter, Robert Voswinckel, Dirk Bandorski, Werner Seeger, Friedrich Grimminger, Hossein-Ardeschir Ghofrani

**Affiliations:** Universities of Giessen & Marburg Lung Center, Medizinische Klinik II, Klinikstraße 33, 35392 Giessen, Germany; Medical Clinic V, University of Munich, Comprehensive Pneumology Center, Munich, Germany; Department of Internal Medicine, Friedberg Hospital, Friedberg, Germany; Max Planck Institute for Heart and Lung Research, Bad Nauheim, Germany; Faculty of Medicine, Imperial College London, London, UK

**Keywords:** Combination therapy, Iloprost, Sildenafil, Pulmonary hypertension, Giessen pulmonary hypertension registry

## Abstract

**Background:**

Combination therapy is frequently used to treat patients with pulmonary hypertension but few studies have compared treatment regimens. This study examined the long-term effect of different combination regimens of inhaled iloprost and oral sildenafil on survival and disease progression.

**Methods:**

This was a retrospective study of patients in the Giessen Pulmonary Hypertension Registry who received iloprost monotherapy followed by addition of sildenafil (iloprost/sildenafil), sildenafil monotherapy followed by addition of iloprost (sildenafil/iloprost), or upfront combination therapy (iloprost + sildenafil). The primary outcome was transplant-free survival (Kaplan–Meier analysis). When available, haemodynamic parameters and 6-minute-walk distance were evaluated.

**Results:**

Overall, 148 patients were included. Baseline characteristics were similar across treatment groups; however, the iloprost + sildenafil cohort had higher mean pulmonary vascular resistance and pulmonary arterial pressure than the others. Transplant-free survival differed significantly between groups (*P* = 0.007, log-rank test). Cumulative transplant-free survival was highest for patients who received iloprost/sildenafil (1 year survival: iloprost/sildenafil, 95.1 %; sildenafil/iloprost, 91.8 %; iloprost + sildenafil, 62.9 %); this group also remained on monotherapy significantly longer than the sildenafil/iloprost group (median 17.0 months vs 7.0 months, respectively; *P* = 0.004). Compared with pre-treatment values, mean 6-minute-walk distance increased significantly for all groups 3 months after beginning combination therapy.

**Conclusions:**

In this observational study of patients with pulmonary hypertension receiving combination therapy with iloprost and sildenafil, cumulative transplant-free survival was highest in those who received iloprost monotherapy initially. However, owing to the size and retrospective design of this study, further research is needed before making firm treatment recommendations.

**Electronic supplementary material:**

The online version of this article (doi:10.1186/s12890-015-0164-2) contains supplementary material, which is available to authorized users.

## Background

Pulmonary hypertension (PH) is a life-threatening disorder with a variety of aetiologies [1]. Because PH is a multifactorial condition, monotherapy focused on a single pathological pathway may be insufficient to halt disease progression. By acting on two or more biological pathways, combination therapies have the potential for increased efficacy over monotherapies. In patients with PH, two main approaches for combining treatments may be followed, with therapies introduced sequentially or concomitantly as ‘upfront’ combination therapy. Monotherapy is normally used initially, with additional therapy introduced if clinical deterioration occurs. Less frequently, combination treatment is used as first-line therapy to exploit the ‘hit hard and early’ model, which aims to use early and aggressive treatment to halt disease progression [2]. This approach is also recommended in international guidelines for patients with PH, for those with severe disease (defined as class IV according to the World Health Organisation functional class system) [3].

Treatment guidelines also suggest combining established pharmacotherapies for patients with PH who do not respond adequately to monotherapy, but do not recommend particular combinations or regimens [3]. During a 3 year study employing pre-defined treatment goals to guide therapeutic decisions, combination therapy was eventually required by almost half of patients initially prescribed monotherapy [4]. Several studies have examined the combination of the prostanoid iloprost and the phosphodiesterase type 5 (PDE-5) inhibitor sildenafil in the treatment of patients with PH. In acute haemodynamic testing, combining these drugs led to a greater reduction in pulmonary vascular resistance (PVR) than each agent alone [5]. Furthermore, patients with pulmonary arterial hypertension (PAH) showed improved exercise capacity and haemodynamics when given sildenafil as an add-on to existing iloprost therapy [6]. Randomized controlled trials directly comparing the efficacy of iloprost and sildenafil have not been undertaken, although a meta-analysis found no significant difference in efficacy between these therapies [7]. The aim of this study was to examine the long-term effect of different combination regimens of inhaled iloprost and oral sildenafil on the survival and disease progression of patients with PH.

## Methods

### Study design

This was an observational study [8] of patients in the Giessen Pulmonary Hypertension Registry, a single-centre registry including more than 2500 patients with newly diagnosed disease. The registry started in 1993. For this study, the patients who met the eligibility criteria had been enrolled from 1993 to 2013. Adult patients who received a combination of inhaled iloprost and oral sildenafil were eligible for inclusion. Patients who received intravenous iloprost or sildenafil, or who had begun treatment with therapies other than iloprost or sildenafil, were excluded. Each patient gave informed consent to participate. The study was approved by the University of Giessen Institutional Review Board (reference number 266/11) and followed the principles of the Declaration of Helsinki.

Three treatment regimens were studied: iloprost monotherapy followed by addition of sildenafil (iloprost/sildenafil); sildenafil monotherapy followed by addition of iloprost (sildenafil/iloprost); and upfront combination therapy of iloprost and sildenafil (iloprost + sildenafil). No pre-defined protocol was followed; treatment and doses were tailored to the individual patient’s needs and optimized by dose-titration.

### Outcome measures

The primary outcome measure was transplant-free survival, as calculated by Kaplan–Meier analysis. As this was a retrospective study of patient records, complete information could not be obtained in all cases. When available, 6-minute-walk distance (6MWD), New York Heart Association (NYHA) functional class, pulmonary arterial pressure (PAP), PVR, and cardiac output were analysed. Changes were compared using intra-individual paired analysis (i.e. including only patients with both baseline and post-treatment data available). Values were determined pre-treatment (baseline), 3 months after monotherapy initiation, before combination therapy initiation (post-monotherapy baseline), and 3 months after combination therapy initiation. Patients lost to follow-up were classified as having withdrawn alive on the date of last contact.

### Statistical methods

Data are presented as mean (standard deviation) or median (interquartile range), as applicable. The log-rank test was used to analyse differences in cumulative transplant-free survival; analysis of variance was applied to test for differences between groups; and the paired *t*-test (two-tailed) was used to examine changes in response to therapy. Cox regression, defining iloprost as the reference, was applied to control for possible confounders in survival analysis, correcting for NYHA functional class, 6MWD, and cardiac output. The Kruskal–Wallis test was performed to test for differences in parameters with skewed distributions.

## Results

### Baseline characteristics

In total, out of 685 patients assessed, 148 patients were eligible for the study. Similar numbers of patients initially received iloprost or sildenafil monotherapy (61 patients and 63 patients, respectively), and 24 received upfront combination therapy (Table 1). In the iloprost/sildenafil group, idiopathic PAH and PAH associated with other conditions (Dana Point classification 1.4) [1] were the most frequent aetiologies (35.0 % and 33.3 %, respectively). Similarly, patients treated with sildenafil/iloprost were mainly those with idiopathic PAH or PAH associated with other conditions (25.0 % and 43.3 %, respectively). The most common classification for patients who received upfront combination therapy was idiopathic PAH (47.8 %). Baseline characteristics were broadly similar in the treatment groups (Table 1). The mean age at diagnosis of the sildenafil/iloprost group was significantly higher than that of the iloprost + sildenafil group (53.0 years vs 43.3 years, respectively; *P* = 0.029); otherwise, there were no significant differences between the mean ages of the groups. Patients who initially received iloprost monotherapy were admitted to the study centre earlier (median date November 2000) than those beginning sildenafil monotherapy or combination therapy (median dates April and August 2003, respectively; *P* < 0.001). The mean baseline 6MWD was lower for patients who received upfront combination therapy than for the other groups (Table 1), but not significantly so (*P* = 0.227).

**Table 1:** Baseline characteristics of patients who were eligible for the observational study

Characteristic	Treatment regimen^a^		
	Iloprost/sildenafil	Sildenafil/iloprost	Iloprost + sildenafil
	*n* = 61	*n* = 63	*n* = 24
Female sex, %	65.0	66.7	78.3
	[*n* = 60]	[*n* = 60]	[*n* = 23]
Mean age at diagnosis, years (SD)	48.7 (14.9)	53.0 (15.2)	43.3 (17.1)
Classification of PH, n (%)			
Idiopathic PAH	21 (35.0)	15 (25.0)	11 (47.8)
PAH associated with other conditions^b^	20 (33.3)	26 (43.3)	6 (26.1)
Associated with lung diseases	4 (6.7)	9 (15.0)	1 (4.3)
CTEPH	14 (23.3)	10 (16.7)	3 (13.0)
Miscellaneous	1 (1.7)	0 (0.0)	2 (8.7)
	[*n* = 60]	[*n* = 60]	[*n* = 23]
NYHA functional class, n (%)			
II	3 (10.3)	4 (8.5)	0 (0)
III	13 (44.8)	18 (38.3)	5 (38.5)
IV	13 (44.8)	25 (53.2)	8 (61.5)
	[*n* = 29]	[*n* = 47]	[*n* = 13]
Mean PAP, mmHg (95% CI)	55 (51–58)	57 (53–61)	73 (65–82)
	[*n* = 50]	[*n* = 51]	[*n* = 21]
Mean cardiac output, L/min (95% CI)	3.4 (3.1–3.7)	3.6 (3.3–3.9)	3.1 (2.5–3.7)
	[*n* = 49]	[*n* = 51]	[*n* = 21]
Mean PVR, dyn.s.cm^−5^ (95% CI)	1287 (1134–1440)	1143 (1016–1270)	1824 (1538–2109)
	[*n* = 49]	[*n* = 51]	[*n* = 21]
Mean 6MWD, m (95% CI)	276 (232–319)	281 (245–317)	222 (179–265)
	[*n* = 38]	[*n* = 48]	[*n* = 16]

*6MWD* 6-minute-walk distance; *CHD* congenital heart disease; *CI* confidence interval; *CTD* connective tissue disease; *CTEPH* chronic thromboembolic pulmonary hypertension; *ILD* interstitial lung disease; *NYHA* New York Heart Association; *PAH* pulmonary arterial hypertension; *PAP* pulmonary arterial pressure; *PVR* pulmonary vascular resistance; *SD* standard deviation

^a^The treatment regimens were: iloprost/sildenafil (iloprost followed by addition of sildenafil), sildenafil/iloprost (sildenafil followed by addition of iloprost), or iloprost + sildenafil (combined iloprost and sildenafil as upfront therapy); ^b^Dana Point classification 1.4 [1]

Patients who received upfront combination therapy had significantly higher mean PAP than patients initially treated with iloprost or sildenafil monotherapy (*P* < 0.001 [Table 1]). Between treatment groups, however, there was no significant difference in cardiac output (*P* = 0.264). Patients treated with upfront combination therapy had higher mean PVR than those who started on iloprost or sildenafil monotherapy (*P* < 0.001). Data for exercise capacity and haemodynamic parameters were not available for all patients. The proportions of patients who went on to receive additional therapy with an endothelin receptor antagonist, an intravenous prostanoid or both were 48.6 %, 5.4 %, and 13.5 %, respectively. Patients were followed up for a mean of 60.9 months.

### Duration of monotherapy treatment

**Fig. 1:**
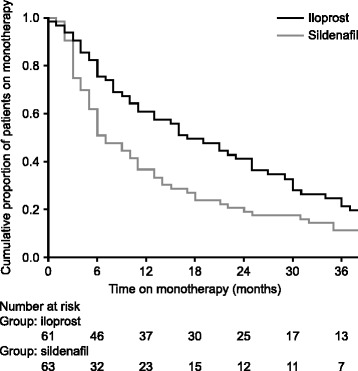
Kaplan–Meier plot of proportions of patients remaining on iloprost or sildenafil monotherapy over time

Patients initially treated with iloprost remained on monotherapy significantly longer than those starting with sildenafil (*P* = 0.004; Fig. 1). Median time on monotherapy was 17.0 months (95 % confidence interval: 10.4–23.6 months) with iloprost and 7.0 months (95 % confidence interval: 4.2–9.8 months) with sildenafil.

### Cumulative transplant-free survival

**Fig. 2:**
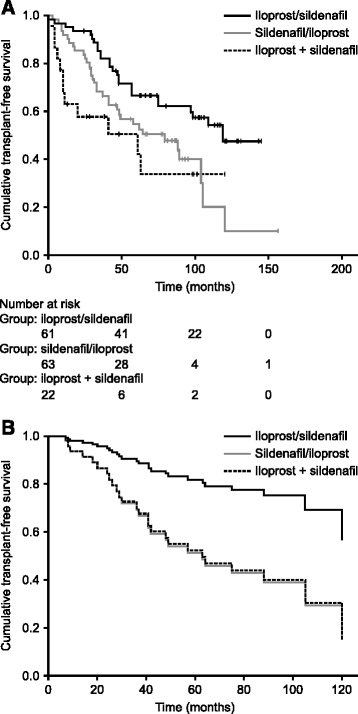
Transplant-free survival. (**a**) Kaplan–Meier plot of cumulative transplant-free survival and (**b**) Cox regression estimate of transplant-free survival after correction for possible confounders (New York Heart Association functional class, 6-minute-walk distance, and cardiac output). Patients were treated sequentially with iloprost and sildenafil (either iloprost followed by addition of sildenafil [iloprost/sildenafil] or sildenafil followed by addition of iloprost [sildenafil/iloprost]), or with upfront combination therapy (iloprost + sildenafil)

In total, eight patients were lost to follow-up: three in the iloprost/sildenafil group, one in the sildenafil/iloprost group, and four in the iloprost + sildenafil group. There was a significant difference in transplant-free survival among groups (*P* = 0.007, log-rank test; Fig. 2a). Cumulative transplant-free survival was highest in the iloprost/sildenafil group and lowest for those who received upfront combination therapy. In the iloprost/sildenafil group, survival rates were 95.1 % at 1 year, 81.8 % at 3 years, and 66.4 % at 5 years. In the sildenafil/iloprost group, survival rates were 91.8 % at 1 year, 68.1 % at 3 years, and 54.5 % at 5 years. Survival rates were 62.9 % at 1 year, 57.7 % at 3 years, and 50.5 % at 5 years for patients who received upfront combination therapy.

After Cox regression analysis, cumulative transplant-free survival was significantly higher in the iloprost/sildenafil group than in the sildenafil/iloprost group (*P* = 0.035; Fig. 2b). Survival was also higher for patients treated with iloprost/sildenafil than for those treated with upfront combination therapy, but this difference was not statistically significant (*P* = 0.120).

### Cumulative transplant-free survival based on the aetiology of pulmonary hypertension

For patients with PAH initially treated with iloprost or sildenafil, cumulative transplant-free survival was analysed by PH classification (Additional file 1: Figure S1). For all groups assessed (PAH associated with collagen-vascular disease, idiopathic PAH, and PAH associated with systemic-to-pulmonary shunt), survival was higher in the iloprost/sildenafil group than in the sildenafil/iloprost group. No statistical analyses were conducted because the number of patients in these sub-analyses was small.

### Change in functional class

**Fig. 3:**
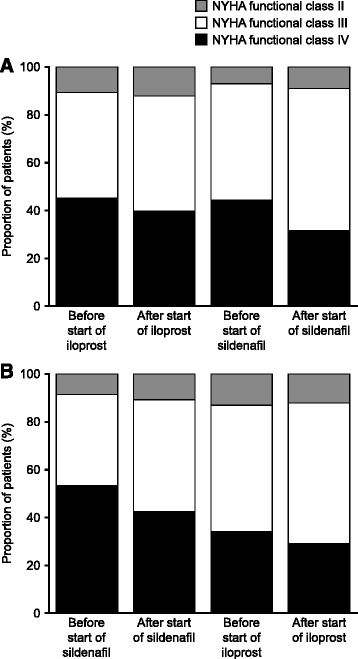
New York Heart Association (NYHA) functional class over the study. **a** Patients received iloprost followed by addition of sildenafil. **b** Patients received sildenafil followed by addition of iloprost

The iloprost/sildenafil group had a lower proportion of patients in NYHA functional class IV at pre-treatment baseline than the sildenafil/iloprost group (Fig. 3). The proportion of patients in NYHA functional class IV showed a more pronounced decrease with sildenafil than with iloprost. The lowest proportion of patients in NYHA functional class IV was observed after addition of the second therapy in both groups.

### Change in mean pulmonary arterial pressure

**Fig. 4:**
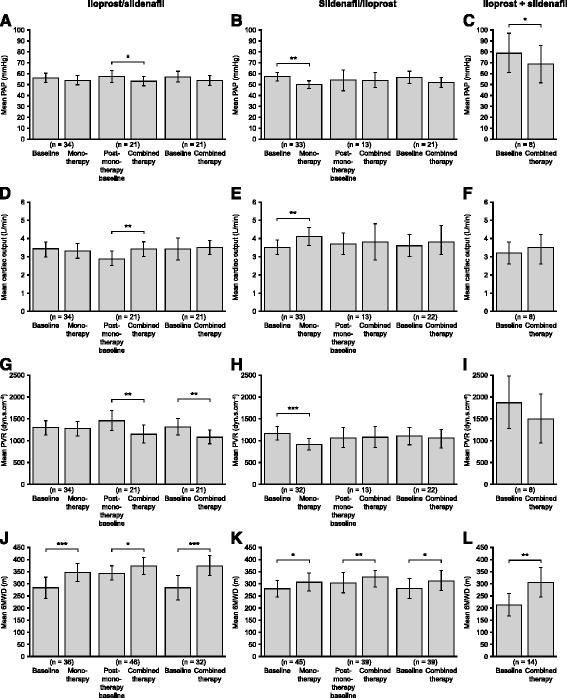
Changes in haemodynamic parameters and 6-minute-walk distance over the study (intra-individual responses). (**a**–**c**) Pulmonary arterial pressure (PAP), (**d**–**f**) cardiac output, (**g**–**i**) pulmonary vascular resistance (PVR), and (**j**–**l**) 6-minute-walk distance (6MWD). Data are presented as means ± 95 % confidence interval. Patients were treated with iloprost followed by addition of sildenafil (iloprost/sildenafil), sildenafil followed by addition of iloprost (sildenafil/iloprost), or upfront combination therapy with iloprost and sildenafil (iloprost + sildenafil). Values are shown pre-treatment (*baseline*), 3 months after therapy initiation (*monotherapy*), before combination therapy (post-monotherapy baseline), and 3 months after starting combination therapy (combined therapy). *P < 0.05; **P < 0.01; ***P < 0.001. Statistical analysis was conducted using the paired sample *t*-test (two-tailed)

There was no significant change in mean PAP measured 3 months after therapy initiation from pre-treatment baseline for patients initially treated with iloprost (Fig. 4a). Following combination therapy, mean PAP was significantly reduced compared with post-monotherapy baseline (*P* = 0.037). However, there was no significant change in mean PAP after 3 months of combination therapy compared with pre-treatment baseline.

Mean PAP was significantly reduced from a pre-treatment baseline of 57 mmHg to 50 mmHg for patients who received sildenafil monotherapy (*P* = 0.001; Fig. 4b). However, mean PAP was unchanged 3 months after beginning combination therapy compared with post-monotherapy baseline. Compared with pre-treatment baseline, there was no significant change in mean PAP after combination therapy (*P* = 0.148).

For patients who began initial combination therapy, mean PAP was significantly reduced from a pre-treatment value of 79 mmHg to 69 mmHg after 3 months of treatment (*P* = 0.018; Fig. 4c).

### Change in mean cardiac output

Mean cardiac output was unchanged 3 months after beginning iloprost therapy compared with pre-treatment values (Fig. 4d). However, after combination therapy, mean cardiac output was increased compared with post-monotherapy baseline, from 2.9 L/min to 3.4 L/min (*P* = 0.001). There was no significant difference between mean cardiac output pre-treatment and following combination therapy.

For patients initially treated with sildenafil, mean cardiac output increased from 3.5 L/min at pre-treatment baseline to 4.1 L/min 3 months after beginning treatment (*P* = 0.001; Fig. 4e). Following iloprost addition, there was no significant change in mean cardiac output compared with post-monotherapy or pre-treatment baselines. Similarly, for patients treated with combination therapy initially, there was no significant change in mean cardiac output compared with pre-treatment baseline (Fig. 4f).

### Change in mean pulmonary vascular resistance

After 3 months of iloprost monotherapy, there was no significant change in mean PVR compared with pre-treatment baseline (Fig. 4g). However, mean PVR was significantly reduced 3 months after initiating combination treatment compared with post-monotherapy baseline, from 1455 dyn.s.cm^−5^ to 1143 dyn.s.cm^−5^ (*P* = 0.006). A significant reduction in mean PVR was also seen following combination therapy when compared with pre-treatment values (*P* = 0.006).

Mean PVR was significantly reduced from a pre-treatment baseline of 1161 dyn.s.cm^−5^ to 909 dyn.s.cm^−5^ 3 months after beginning sildenafil monotherapy (*P* < 0.001; Fig. 4h). However, 3 months after beginning combination therapy there was no change in mean PVR compared with post-monotherapy or pre-treatment baselines.

For patients treated initially with combination therapy, there was no significant change in mean PVR compared with pre-treatment baseline (Fig. 4i).

### Change in 6-minute-walk distance

Compared with pre-treatment values, patients who received initial iloprost monotherapy showed significantly increased mean 6MWD, from 283 m to 346 m (*P* < 0.001; Fig. 4j). Exercise capacity was also improved following add-on sildenafil therapy: compared with post-monotherapy baseline, mean 6MWD increased from 345 m to 374 m (*P* = 0.01). Mean 6MWD increased from 283 m at pre-treatment baseline to 374 m 3 months after beginning combination therapy (*P* < 0.001).

For patients who received initial sildenafil monotherapy, mean 6MWD increased from 278 m at pre-treatment baseline to 307 m 3 months after beginning treatment (*P* = 0.036; Fig. 4k). Subsequently, patients treated with add-on iloprost therapy showed increased exercise capacity compared with post-monotherapy baseline, with mean 6MWD increased from 303 m to 328 m (*P* = 0.002). Compared with pre-treatment values, mean 6MWD increased from 280 m to 312 m for patients treated with combination therapy (*P* = 0.038).

6MWD increased from 213 m to 305 m for patients treated with upfront combination therapy compared with pre-treatment baseline (*P* = 0.001; Fig. 4l).

## Discussion

In the treatment of patients with PH, clinical studies have evaluated combinations of major pharmacological classes of medical therapies, i.e. endothelin receptor antagonists and prostanoids [9–12], endothelin receptor antagonists and PDE-5 inhibitors [13, 14], and prostanoids and PDE-5 inhibitors [5, 6, 15–19]. However, only one study, of administration of the prostanoid treprostinil for up to 2 years in patients receiving oral background PAH therapy, examined long-term outcomes (survival and clinical worsening [defined as addition of a new PAH therapy, discontinuation due to disease progression, or death]) [20]. A meta-analysis of randomized controlled studies in patients with PAH found that, compared with monotherapy, combination therapy significantly reduced clinical deterioration, increased 6MWD, and improved haemodynamics [21]. However, no significant difference in mortality was observed between patients treated with monotherapy and those receiving combination therapy.

Iloprost and sildenafil act via different pathways (stimulating cyclic adenosine monophosphate [cAMP] production and preventing cyclic guanosine monophosphate [cGMP] breakdown, respectively), but there is evidence of cross-talk between these pathways. Raised cGMP levels inhibit cAMP breakdown, and pre-treatment of erythrocytes in vitro with PDE-5 inhibitors potentiated cAMP release in response to treprostinil [22]. In acute haemodynamic testing in patients with PH, the combination of sildenafil and iloprost produced a greater vasodilatory response than either agent alone [5]. There are limited data showing the long-term benefits of combining sildenafil and iloprost, but in a 16 week study the addition of sildenafil to long-term treatment with the prostacyclin epoprostenol improved exercise capacity and haemodynamics among patients with PAH compared with those receiving placebo [23].

In our study, cumulative transplant-free survival was lower for patients who received upfront combination therapy than for those treated with initial monotherapy. At 1 year, the survival rate was 62.9 % for those who received combination therapy, compared with 95.1 % and 91.8 % for those first treated with inhaled iloprost or oral sildenafil monotherapy, respectively. However, before therapy, patients treated with iloprost + sildenafil had higher mean PVR and mean PAP than those who began monotherapy. Therefore, patients with the most severe disease had been assigned to receive upfront combination therapy, as is recommended in current international treatment guidelines for patients with PH [3]. This approach, of treating patients with severe PAH with upfront inhaled iloprost and oral sildenafil therapy, was taken in a separate study of eight patients of NYHA functional class IV who were unable to perform a 6MWD test. Following treatment, all patients had an improvement in their functional class and were able to complete a 6MWD test, though one patient later underwent lung transplantation and subsequently died [24]. Similarly, for the small number of patients for whom measurements were recorded in our study (*n* = 16), 6MWD significantly increased following combination treatment.

Among patients treated initially with monotherapy, transplant-free survival was higher for those receiving iloprost/sildenafil than for those treated with sildenafil/iloprost. Patients treated with inhaled iloprost also remained on monotherapy longer than patients beginning oral sildenafil monotherapy. When paired recordings were available, the benefit of sequential therapy on exercise capacity was also observed for both drug regimens, with 6MWD significantly higher than pre-treatment values after 3 months of combination therapy. The results from this study suggest that when combining iloprost and sildenafil in a step-wise manner the optimal treatment regimen may be initial monotherapy with iloprost followed by add-on sildenafil if clinical deterioration occurs.

This study has limitations. Owing to the retrospective design, patients were not randomly assigned to each treatment, as highlighted by significant differences in baseline characteristics between the patient groups. Furthermore, complete data were not available for functional class, haemodynamic parameters, and exercise capacity, and there was the potential for selection bias. Unlike treatment with iloprost monotherapy, sildenafil monotherapy resulted in significant improvements in haemodynamics compared with pre-treatment values. Despite this, transplant-free survival was shorter in patients initially treated with sildenafil than among those who received iloprost therapy first. This is difficult to explain, and may reflect differences in the baseline characteristics between these patient groups or be the result of an unrecognized confounding factor. For these reasons, caution is needed when comparing the effectiveness of these two monotherapies. These outcomes also need to be viewed in the context of treatment practices over the course of this study, because patients who received initial iloprost therapy were admitted to the study centre before those first treated with sildenafil. In Europe, iloprost was approved for the treatment of PAH 2 years before sildenafil. Thus, in the early years of the study, inhaled iloprost was the only treatment available and patients may have remained on monotherapy because no other treatment options were available. Furthermore, this study included patients from a period of 20 years, over which time there were significant changes in diagnostic practices and in the clinical management of PH, which may have influenced the outcomes of patients.

## Conclusions

In this observational study, the sequence in which patients with PH received combination therapy with iloprost and sildenafil was independently associated with transplant-free survival rate. However, owing to the small size of the study and its retrospective design, further research is required to confirm the external validity of the results.

### Availability of Data and Materials

Data are available from the corresponding author upon request.

## Additional file

Additional file 1: Figure S1. Kaplan–Meier plots of cumulative transplant-free survival in patients with pulmonary arterial hypertension associated with collagen-vascular disease, idiopathic pulmonary arterial hypertension, and pulmonary arterial hypertension associated with systemic-to-pulmonary shunt. Data are shown for patients who were treated with iloprost followed by addition of sildenafil (iloprost/sildenafil) or sildenafil followed by addition of iloprost (sildenafil/iloprost). (PDF 853 kb)

## Abbreviations

cAMP: Cyclic adenosine monophosphate
cGMP: Cyclic guanosine monophosphate
6MWD: 6-minute-walk distance
NYHA: New York Heart Association
PAH: Pulmonary arterial hypertension
PAP: Pulmonary arterial pressure
PDE-5: Phosphodiesterase type 5
PH: Pulmonary hypertension
PVR: Pulmonary vascular resistance
