# Supplementary material for: Survival with sildenafil and inhaled iloprost in a cohort with pulmonary hypertension: an observational study
Source: BMC Pulm Med. 2016 Jan 12;16:5. doi: 10.1186/s12890-015-0164-2 (PMC4709958; doi:10.1186/s12890-015-0164-2)
Supplement: Additional file 1: Figure S1. — Kaplan–Meier plots of cumulative transplant-free survival in patients with pulmonary arterial hypertension associated with collagen-vascular disease, idiopathic pulmonary arterial hypertension, and pulmonary arterial hypertension associated with systemic-to-pulmonary shunt. Data are shown for patients who were treated with iloprost followed by addition of sildenafil (iloprost/sildenafil) or sildenafil followed by addition of iloprost (sildenafil/iloprost). (PDF 853 kb) [file 12890_2015_164_MOESM1_ESM.pdf]

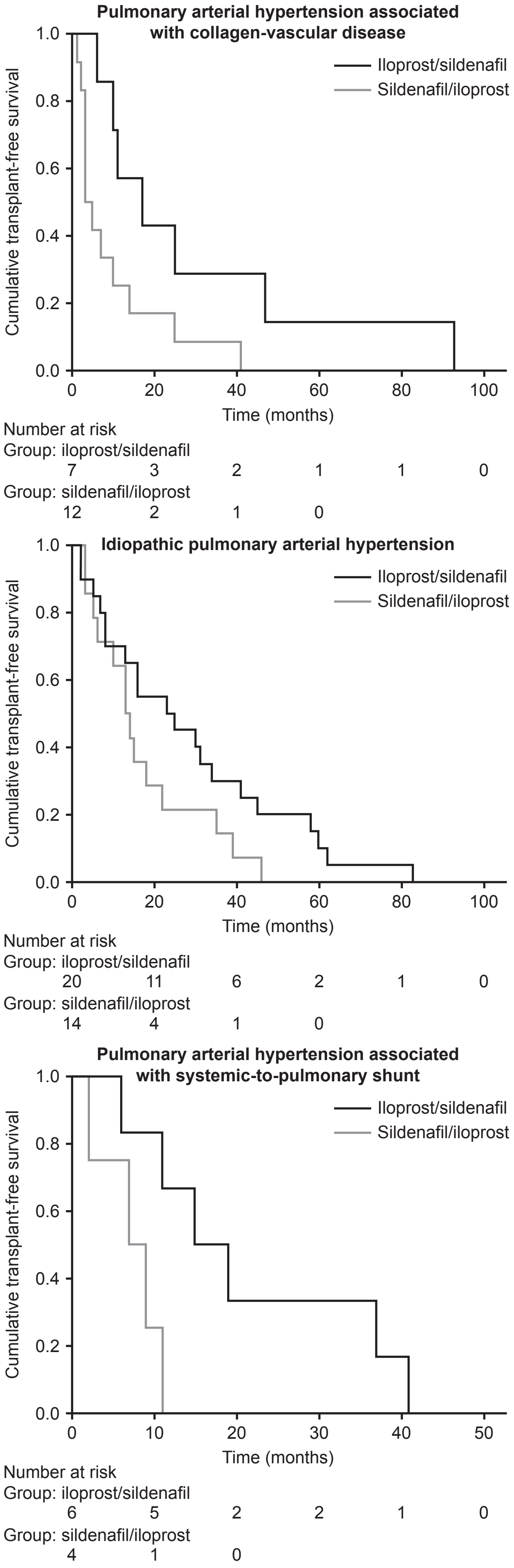

Pulmonary arterial hypertension associated with collagen-vascular disease
Cumulative transplant-free survival
1.0
0.8
0.6
0.4
0.2
0.0
0
20
40
60
80
100
Time (months)
Iloprost/sildenafil
Sildenafil/iloprost
Number at risk
Group: iloprost/sildenafil
7 3 2 1 1 0
Group: sildenafil/iloprost
12 2 1 0
Idiopathic pulmonary arterial hypertension
Cumulative transplant-free survival
1.0
0.8
0.6
0.4
0.2
0.0
0
20
40
60
80
100
Time (months)
Iloprost/sildenafil
Sildenafil/iloprost
Number at risk
Group: iloprost/sildenafil
20 11 6 2 1 0
Group: sildenafil/iloprost
14 4 1 0
Pulmonary arterial hypertension associated with systemic-to-pulmonary shunt
Cumulative transplant-free survival
1.0
0.8
0.6
0.4
0.2
0.0
0
10
20
30
40
50
Time (months)
Iloprost/sildenafil
Sildenafil/iloprost
Number at risk
Group: iloprost/sildenafil
6 5 2 2 1 0
Group: sildenafil/iloprost
4 1 0
